# Outbreak of penicillin-susceptible, *eta*-positive *Staphylococcus aureus* in a neonatal intensive care unit

**DOI:** 10.3389/fmicb.2026.1946607

**Published:** 2026-10-27

**Authors:** David Meintrup, Daniel Vilser, Franziska Layer-Nicolaou, Fabian Müller, Birgit Strommenger, Beate Rieß, Brigitte Hücherig, Stefan Borgmann

**Affiliations:** 1 Faculty of Engineering and Management, University of Applied Sciences Ingolstadt, Ingolstadt, Germany; 2 Department of Pediatrics and Adolescent Medicine, AMEOS St. Elisabeth Clinic, Neuburg an der Donau, Germany; 3 Robert Koch Institute, Wernigerode, Germany; 4 Department of Infectious Diseases and Infection Control, Ingolstadt Hospital, Ingolstadt, Germany; 5 Clinic St. Hedwig, Regensburg, Germany

**Keywords:** exfoliative toxin A (ETA), neonatal intensive care unit (NICU), penicillin-susceptible, purulent conjunctivitis, screening, ST15, *Staphylococcus aureus*

## Abstract

*Staphylococcus aureus* is one of the most common microorganisms causing wound infections, and outbreaks of multi-drug-resistant strains in neonatal intensive care units (NICUs) are a major concern. In Germany, screening patients for colonization with multi-drug-resistant bacteria is strongly recommended in these wards under national infection-control guidelines. Outbreaks caused by penicillin-susceptible strains are less commonly reported. In the first few months of 2024, penicillin-susceptible *S. aureus* was detected in 20 infants, of whom 10 developed confirmed infections, primarily purulent conjunctivitis. Using a cumulative sum (CUSUM) control chart applied to monthly acquisition counts from January 2021 to December 2025, an outbreak was identified between December 2023 and May 2024. Whilst the detection rate during the non-outbreak phase was 1.17 patients per month, it rose to 3.33 patients per month during the outbreak (*p* < 0.001). The number of infections was also significantly higher, with 1.67 infections per month within the outbreak period compared to 0.30 infections per month (*p* < 0.001). The median gestational age was lower in patients with a confirmed infection than in patients with colonization only (214.5 vs. 243.5 days, *p* = 0.029). Four isolates from three affected children had undergone detailed typing. All isolates exhibited the same *spa*- (t328), ST- (15), and CT- (38152) types. In the cgMLST analysis, these isolates form a single cluster. All four isolates harbored the *eta* gene, suggesting the potential for expression of exfoliative toxin A. Following implementation of a bundle of enhanced infection-control measures, no further outbreak-associated detections occurred. As part of this response, and because systematic identifiable staff screening was restricted by data-protection requirements, all staff members were offered voluntary anonymous screening for nasal *S. aureus* colonization and, where appropriate, access to mupirocin decolonization. Although the contribution of individual interventions cannot be determined, the temporal association suggests that the combined infection-control measures likely contributed to containment of the outbreak. Our report shows that antibiotic susceptible pathogens can cause outbreaks in NICUs. Surveillance that also includes susceptible pathogens may facilitate the detection and epidemiological assessment of suspicious clusters.

## Introduction

*Staphylococcus aureus* is an important cause of healthcare-associated infections in neonates, particularly in preterm infants. Infection-control surveillance in neonatal intensive care units (NICUs) has traditionally focused on multi-drug-resistant organisms, especially methicillin-resistant *S. aureus* (MRSA). However, methicillin-susceptible *S. aureus* (MSSA), including penicillin-susceptible strains, may also be transmitted within NICUs and cause clinically relevant outbreaks. Compared with MRSA, transmission of susceptible *S. aureus* strains has been less extensively characterized. In addition to antimicrobial resistance, the pathogenic potential of *S. aureus* is increased by virulence factors. Exfoliative toxins (ETs) are serine proteases that cleave desmoglein-1 and may cause localized bullous impetigo or, when toxin effects become systemic, staphylococcal scalded skin syndrome (SSSS) (Bukowski et al., 2010; Simpson et al., 2010). *S. aureus* most commonly produces exfoliative toxin A (ETA) and B (ETB). Neonates, particularly preterm infants, are especially susceptible to disease caused by ET-producing *S. aureus*. Antibody production increases with gestational age, which may contribute to the greater vulnerability of very preterm infants (Saida et al., 2015). ETA- and ETB-producing *S. aureus* strains have been associated with community spread and healthcare-associated outbreaks (Lamanna et al., 2017; Pimentel de Araujo et al., 2018; Vendrik et al., 2022; Roer et al., 2025). Recognition of outbreaks caused by susceptible *S. aureus* may be difficult because individual detections can occur weeks apart and susceptible isolates are not necessarily retained for molecular analysis. Molecular typing can therefore be important for distinguishing sporadic detections from transmission events. Whole-genome sequencing and core-genome multi-locus sequence typing (cgMLST) provide high-resolution assessment of genetic relatedness and can complement epidemiological surveillance (Leopold et al., 2014).

In this study, we investigated an outbreak of penicillin-susceptible *S. aureus* in a NICU in South-East Germany that was recognized through ongoing routine microbiological surveillance. The outbreak was characterized by an increased number of *S. aureus* detections and clinical infections, particularly purulent conjunctivitis and wound infections. Molecular characterization of available isolates identified a closely related *eta*-positive ST15 lineage. We combined epidemiological surveillance, genomic typing, and infection-control investigations to characterize the event and its temporal extent. This outbreak adds to the limited literature on transmission of antimicrobial-susceptible, toxin-producing *S. aureus* in neonatal intensive care settings and highlights that clinically relevant transmission may occur even in the absence of antimicrobial resistance.

## Materials and methods

### Study setting and study design

This outbreak investigation was conducted in the neonatal intensive care unit (NICU) at Ingolstadt Hospital, Germany, operated by the Department of Pediatrics and Adolescent Medicine of AMEOS Klinikum St. Elisabeth Neuburg. The 11-bed unit treats approximately 150 patients annually, including extremely preterm infants from 23+0 weeks of gestation. The investigation combined ongoing routine microbiological surveillance with retrospective epidemiological analysis. Screening results are reviewed weekly by the pediatric and infection-control teams, including findings of antimicrobial-susceptible potentially pathogenic organisms. Relevant clusters trigger additional investigations and infection-control measures. During routine surveillance in late 2023 and early 2024, an increase in detections of penicillin-susceptible *Staphylococcus aureus*, including several clinical infections, was recognized. This prompted targeted environmental investigations and additional infection-control measures, including voluntary staff screening. To define the temporal extent of the event and compare detection rates with preceding and subsequent periods, microbiological records from January 2021 through December 2025 were retrospectively analyzed. Only the first detection of penicillin-susceptible *S. aureus* per patient was included. Clinical records, laboratory data, and documentation from weekly infection-control meetings were reviewed to classify detections as colonization, possible infection, or confirmed infection. Laboratory findings were transferred from LabCentre l.i.c. (Version 202601, Mesalvo, Mannheim, Germany) to Hybase (Hybase Statistik V.6.2025.04.01, epiNET GmbH, Bochum, Germany), which permits retrospective extraction according to organism and antimicrobial-susceptibility profile.

### Surveillance screening and case definitions

All NICU patients underwent routine surveillance once weekly using swabs from the axillae, nose, ear, and rectum. Additional samples were obtained when clinically indicated, including from wounds, suspected skin infections, or the affected eye in patients with conjunctivitis. Screening frequency and routine sampling sites remained unchanged during and after the outbreak. Although routine neonatal surveillance primarily targets multi-drug-resistant organisms, the culture procedures also detect susceptible potentially pathogenic organisms, including *S. aureus*. These findings are routinely reported and reviewed and may lead to infection-control measures. Penicillin-susceptible *S. aureus* was therefore detectable within the same routine surveillance system throughout the study period and was not searched for only after recognition of the outbreak. Colonization was defined as detection of penicillin-susceptible *S. aureus* in a surveillance sample without clinical evidence of infection, relevant inflammatory abnormalities, or antimicrobial treatment attributable to *S. aureus*. Possible infection was defined as a clinical condition requiring antimicrobial treatment in which a causal relationship to the detected *S. aureus* could not be established. Confirmed infection was defined as isolation of penicillin-susceptible *S. aureus* from a clinically infected site, such as a wound or an affected eye in purulent conjunctivitis, or from blood culture with compatible clinical findings. If a child was initially found to be colonized but subsequently developed an infection, the finding was classified as an infection, and the date of infection was recorded.

### Microbiological procedures

Screening swabs were cultured on Columbia blood agar containing 5% sheep blood (bioMérieux, Nürtingen, Germany). When testing was explicitly restricted to MRSA, ChromID MRSA agar (bioMérieux) was used. Typical colonies were identified as *S. aureus* using the Pasteurex Staph-Plus test (Bio-Rad, Feldkirchen, Germany), MALDI-TOF MS with the Vitek MS system, or Vitek 2 Compact (bioMérieux). Antimicrobial susceptibility was determined using the Vitek 2 Compact AST P654 card. Environmental contact samples were cultured on caso agar with neutralizing agents (PO5172C; Oxoid, Wesel, Germany), subcultured on Columbia blood agar, and identified as described above. To assess possible changes in background *S. aureus* epidemiology or sampling intensity, monthly numbers of penicillin-resistant *S. aureus* detections, swabs, blood cultures, and catheter-tip cultures were also extracted. For penicillin-resistant *S. aureus*, only the first detection per patient was counted.

#### Molecular characterization

Penicillin-susceptible *S. aureus* isolates were not routinely archived before the outbreak investigation; consequently, isolates from most epidemiologically identified cases were unavailable for retrospective molecular analysis. Four available isolates from three patients sampled during the central outbreak period were submitted to the German National Reference Centre for Staphylococci and Enterococci (NRC). Two isolates from one patient were included because the colonies differed macroscopically. Two additional isolates collected at the end of the epidemiologically defined outbreak period and shortly thereafter were submitted for molecular characterization because continued transmission of the *eta*-positive outbreak strain was suspected. At the NRC, isolates were cultured on sheep blood agar and confirmed as *S. aureus* by clumping-factor detection and tube coagulase testing. Genomic DNA was extracted from overnight cultures using the DNeasy Tissue Kit (Qiagen, Hilden, Germany), with lysostaphin added during cell lysis. *mecA*, *eta*, and *etb* were detected by PCR (Murakami et al., 1991; Jarraud et al., 2002).

Whole-genome sequencing libraries were prepared using the Nextera XT DNA Library Prep Kit (Illumina, San Diego, CA, USA) and sequenced on an Illumina NextSeq 550 using 2 × 150-bp paired-end reads. Read quality was assessed with FastQC and reads were assembled using SKESA. MLST and cgMLST were determined using SeqSphere+ (Ridom GmbH, Germany) according to published schemes (Enright et al., 2002; Leopold et al., 2014). A threshold of ≤10 allelic differences defined a cgMLST cluster. *bla*Z was identified in assembled genomes using BLAST within SeqSphere+ and an in-house resistance-gene database. Because only a subset of isolates was available, molecular findings established relatedness among characterized isolates but could not assign every epidemiological case to the outbreak clone.

### Infection-control analysis

Routine environmental surveillance included 14 contact surface samples twice yearly, generally in January and July, and annual sampling of five crocheted toys used by patients and reprocessed externally. After the increase in *S. aureus* detections and infections, 10 patient-environment samples and five reprocessed incubators were collected in February and April 2024. Three plaster strips from one affected patient and ten samples from touch surfaces were also examined for a possible environmental reservoir. Voluntary anonymous nasal screening for *S. aureus* was offered to all NICU staff between June 20 and July 28, 2024. Because identifiable systematic staff screening was restricted by data-protection requirements, results were accessible only to the respective staff member by individual code. Staff with a positive result could anonymously obtain mupirocin-containing nasal ointment, and repeat testing was possible. Because staff screening was anonymous and staff isolates were not molecularly compared with patient isolates, staff-to-patient or patient-to-staff transmission could not be determined.

### Statistical analysis

Monthly numbers of patients with a first detection of penicillin-susceptible *S. aureus* were calculated for January 2021 through December 2025. A cumulative sum (CUSUM) control chart identified an unusual increase in detections and defined the outbreak period as December 2023 through May 2024. Monthly detection and infection rates during and outside this period were compared using Poisson regression. Infection proportions were compared using one-sided Fisher’s exact test. Pearson’s correlation coefficient was used to assess associations between monthly penicillin-susceptible *S. aureus* detections and monthly counts of penicillin-resistant *S. aureus*, swabs, blood cultures, and catheter-tip cultures. Gestational age at birth was converted to days. Because of small group sizes and non-normal distributions, colonization and confirmed-infection groups were compared using the Mann–Whitney U test. No inferential comparison involving the possible-infection group was performed because it comprised only two patients. Additional statistical details are provided in the Supplementary material.

### Ethics statement

The research analysis presented here was based on retrospective evaluation of routinely collected clinical and microbiological data in anonymized form. Infection-control investigations and measures undertaken during the outbreak were part of routine clinical outbreak management and were not implemented as research interventions. Following a request submitted on February 27, 2026, the Ethics Committee of the Bavarian State Medical Association confirmed on March 3, 2026, that formal ethics approval was not required for the retrospective analysis of existing anonymized data.

## Results

### Epidemiological characteristics of the outbreak

Between December 2023 and May 2024, penicillin-susceptible *S. aureus* was detected in 20 patients in our NICU. Table 1 shows the properties of the patients affected during this period. Ten patients had a confirmed infection, two were classified as having a possible infection, and eight had colonization without clinical infection. Among the ten patients with confirmed infection, eight had purulent conjunctivitis. Four patients had wound infections, including two who also had conjunctivitis. Six of the eight patients with conjunctivitis were younger than 28 days at the time of detection.

**TABLE 1:** Characteristics of patients in the NICU from whom Penicillin susceptible *S. aureus* was isolated during the outbreak period (December 2023 to May 2024).

No.	Sex	Sampling date	Age (days)	Body site	Gestational age at birth	Mode of delivery	Type of sectio
Infection							
2	F	9 Dec 2023	6	Eye	30+5	Sectio	Secondary
5	M	5 Jan 2024	66	Eye	24+5	Sectio	Secondary
6	F	21 Jan 2024	8	Eye, Wound, Ear and Mouth	31+1	Sectio	Primary
8	F	29 Jan 2024	4	Wound	31+0	Sectio	Secondary
9	M	31 Jan 2024	6	Wound Navel	31+1	Sectio	Secondary
12	M	11 Mar 2024	4	Eye	29+0	Sectio	Primary
13	F	8 Apr 2024	7	Eye	30+4	Sectio	Secondary
15	F	10 Apr 2024	14	Eye	41+3	Sectio	Primary
16	F	15 Apr 2024	14	Eye, Wound	30+4	Sectio	Secondary
20	M	27 May 2024	37	Eye	25+1	Sectio	Primary
Possible Infection							
1	M	4 Dec 2023	11	Screening	25+6	Sectio	Primary
4	M	8 Dec 2023	10	Screening	27+4	Sectio	Secondary
No Infection							
3	M	11 Dec 2023	39	Screening	37+0	Sectio	Primary
7	F	22 Jan 2024	13	Screening	32+0	Sectio	Primary
10	M	26 Feb 2024	6	Screening	39+6	SD	
11	F	4 Mar 2024	10	Screening	31+6	Sectio	Primary
14	M	8 Apr 2024	9	Screening	27+5	Sectio	Primary
17	F	25 Apr 2024	54	Screening	32+4	SD	
18	M	7 May 2024	31	Screening	40+1	SD	
19	F	27 May 2024	4	Screening	37+5	Sectio	Primary
X	M	3 Jun 2024	10	Screening	29+1	Sectio	Emergency

The patients were numbered chronologically in the first column. Although the isolate from patient X was collected outside the outbreak period (June 2024), it is also shown due to the results of molecular typing. Age (Days), Age of patients at the time of initial *S. aureus* detection; NA, no assignment; ND, not done; SD, spontaneous delivery; Sectio, caesarean section.

The monthly detection rate increased from 1.17 patients per month outside the outbreak period to 3.33 patients per month during the 6-month outbreak period (Poisson regression, *p* < 0.001; Figure 1A). Similarly, ten confirmed infections occurred during the outbreak period (1.67 per month), compared with 16 infections during the remaining 54 months of the observation period (0.30 per month; *p* < 0.001; Figure 1B).

**FIGURE 1:**
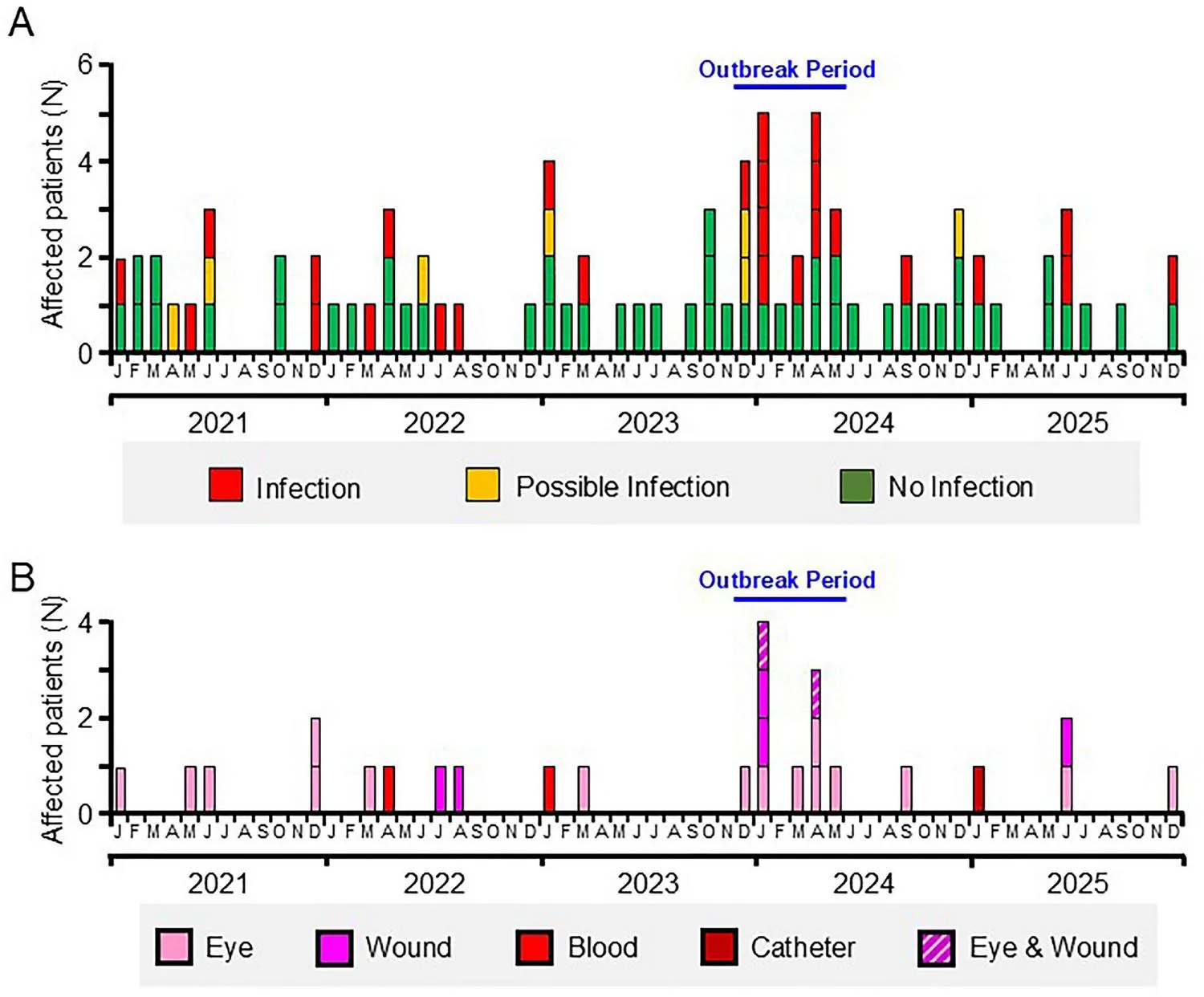
Number of patients per month in the period 2021–2025 in a NICU exhibiting penicillin-susceptible *S. aureus*. **(A)** Monthly number of all affected patients, differentiating to whether there was infection, possible infection or colonization caused by the bacteria. **(B)** Differentiation of infections according to the respective sampling location. The light blue lines at the top of panels **(A,B)** indicate the outbreak period.

During the outbreak period, 10 of 20 detections (50%) were classified as confirmed infections, compared with 16 of 63 detections (25.4%) outside the outbreak period. The corresponding odds ratio was 2.94, with *p*-values ranging from 0.032 to 0.039 depending on the classification of ambiguous cases. Infection proportions before and after the outbreak were similar (26.2% and 23.8%, respectively; Supplementary Figure 1).

### Molecular characterization of available isolates

Four isolates from three patients sampled during the central outbreak period were available for detailed molecular characterization. Two isolates were obtained from the umbilical wound of patient 9 because colonies showed different macroscopic appearances on the primary culture plate. The remaining isolates originated from conjunctival samples from patients 12 and 13. All four isolates had identical spa type t328, sequence type ST15, and cgMLST complex type 38152 and formed a single cgMLST cluster (Table 2). All carried the *eta* gene and lacked *mecA* and *blaZ*. Allelic distances between the four isolates ranged from 0 to 7 alleles, consistent with their close genomic relatedness.

**TABLE 2:** Molecular characterization of *S. aureus* isolates sent to the NRC.

No.	*bla*Z	*mecA*	*eta*	*etb*	*spa*-type	MLST	cgMLST	Cluster
9a	−	−	+	−	t328	ST15	38152	1
9b	−	−	+	−	t328	ST15	38152	1
12	−	−	+	−	t328	ST15	38152	1
13	−	−	+	−	t328	ST15	38152	1
20	−	−	−	−	t049	ST45	38914	None
X	−	−	−	−	t084	ST582	38917	None

The numbers in the first column correspond to the patient numbers in Table 1.

Two additional isolates obtained later were genetically distinct. The isolate from patient 20, collected on May 27, 2024, was spa type t049/ST45/cgMLST 38914, whereas the isolate from patient X, collected on June 3, 2024, was spa type t084/ST582/cgMLST 38917. Neither carried *eta*. Both differed from the ST15 cluster by more than 300 alleles (Figure 2), indicating that they were not part of the characterized outbreak cluster.

**FIGURE 2:**
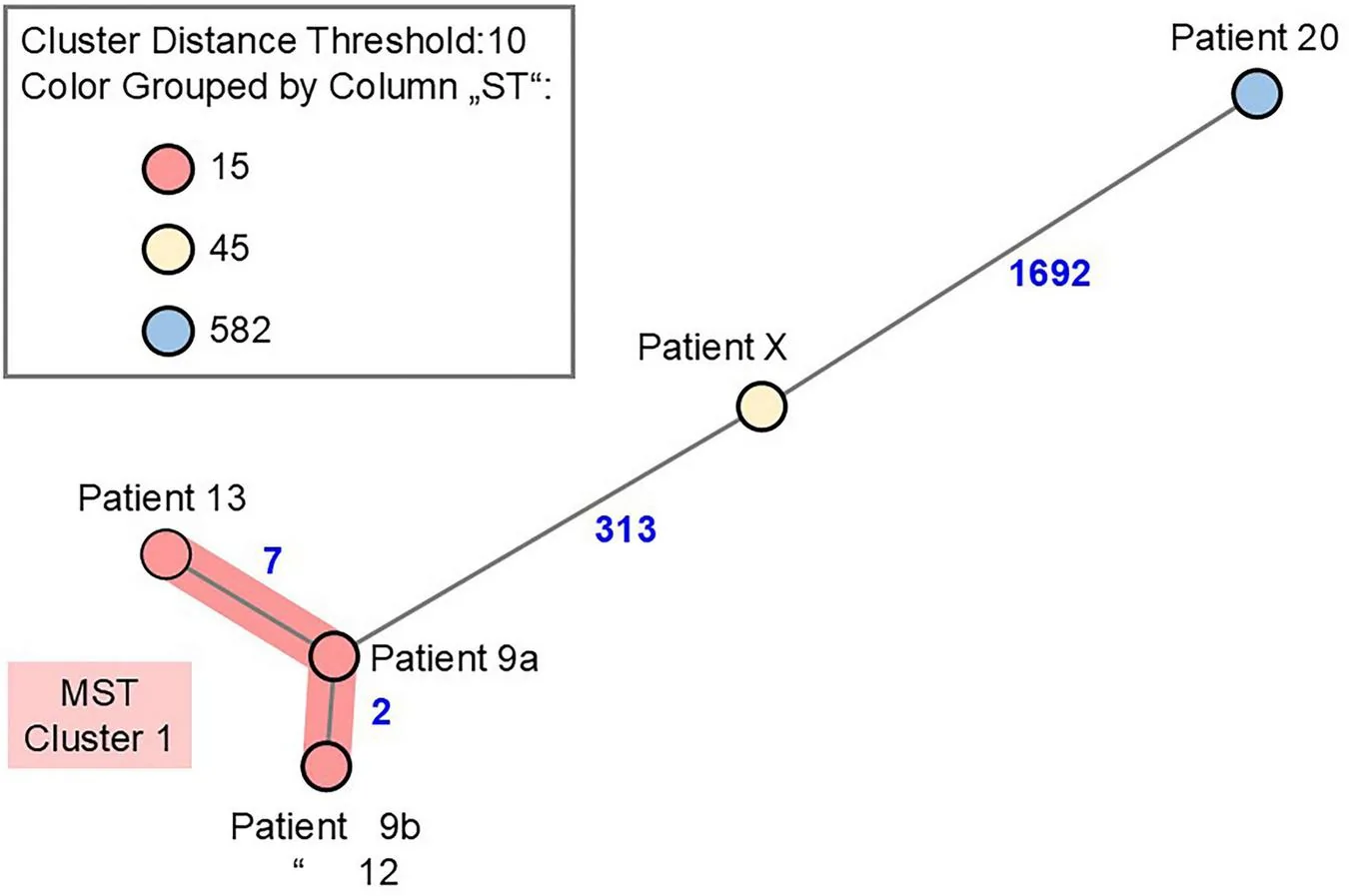
Minimum spanning tree for six samples based on 1781 columns, pairwise ignoring missing values. Nodes are colored by the respective sequence type (ST) according to MLST. Isolates from patient 9, 12 and 13 differed by only 0 – 7 alleles (bold blue numbers), showing a close relationship and indicating transmission events. Isolates from patient 20 and X, isolated later, are differing in >300 alleles from the cluster 1 and have been acquired independently.

### Gestational age and clinical manifestation

Gestational age differed between patients with colonization and those with confirmed infection. Median gestational age was 243.5 days (34.8 weeks; *n* = 8) in patients with colonization only and 214.5 days (30.6 weeks; *n* = 10) in patients with confirmed infection (Mann–Whitney U test, *p* = 0.029). The two patients classified as possible infections had gestational ages of 181 and 193 days, corresponding to a median of 187 days (26.7 weeks); because of the small group size, no inferential comparison involving this group was performed (Supplementary Figure 2).

#### Assessment of surveillance intensity and potential confounding

Monthly detections of penicillin-susceptible *S. aureus* showed little correlation with detections of penicillin-resistant *S. aureus* (Pearson’s *R* = 0.14; Supplementary Figure 3). Correlations with the monthly numbers of microbiological specimens were likewise low: *R* = 0.30 for swabs, *R* = 0.12 for blood cultures, and *R* = 0.21 for catheter-tip cultures (Supplementary Figure 4).

#### Infection-control investigations

As shown in Supplementary Table 1 environmental investigations did not identify *S. aureus* in the sampled locations or materials. This analysis included samples from the environment of an affected patient, reprocessed incubators, samples from touch surfaces, and plaster strips associated with another affected patient.

Voluntary anonymous nasal staff screening was performed between June 20 and July 28, 2024. A total of 57 screening samples were analyzed. As shown by Figure 3 *S. aureus* was not detected in 43 samples; penicillin-susceptible *S. aureus* was detected in seven and penicillin-resistant *S. aureus* in seven samples. Staff members with a positive screening result could anonymously obtain mupirocin-containing nasal ointment.

**FIGURE 3:**
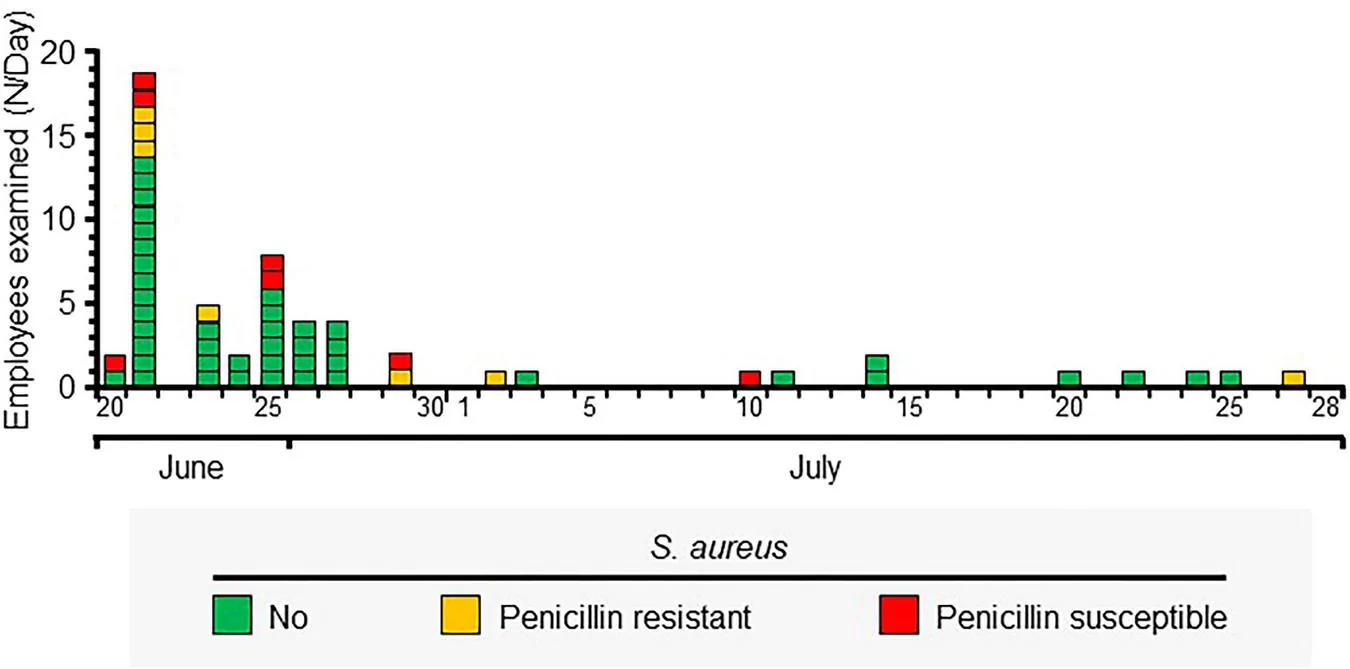
Number of nasal screening samples collected per day from NICU staff during voluntary anonymous *S. aureus* screening. No screening sample was positive for methicillin-resistant *S. aureus*. Observation period: 20 June to 28 July 2024.

Of the seven detections of penicillin-susceptible *S. aureus*, five occurred within the first 6 days of staff screening, one on day 10, and one on day 21. No further penicillin-susceptible *S. aureus* was detected during the final two and a half weeks of the staff-screening period.

## Discussion

The current study describes an outbreak of penicillin-susceptible *S. aureus* in a NICU. Between December 2023 and May 2024, both the number of detections and the number of clinical infections increased substantially compared with the remaining observation period. Penicillin-susceptible *S. aureus* was detected in 20 patients, of whom ten developed confirmed infections. Eight of these infections presented as purulent conjunctivitis, and four patients had wound infections. Molecular characterization of four available isolates from three patients demonstrated a closely related *eta*-positive ST15 cluster, supporting transmission of this strain within the NICU. Another factor suggesting nosocomial transmission is the fact that all of the children suffering from conjunctivitis were born by caesarean section, which in turn makes mother-to-child transmission during vaginal delivery unlikely. Staphylococci are among the microorganisms commonly associated with neonatal conjunctivitis (Butt et al., 2018; Nelson et al., 2025; Turgut et al., 2026). In our cohort, the infections remained localized and responded well to topical antibiotic treatment. Nevertheless, *S. aureus* is also an important cause of severe and systemic infections in preterm infants and neonates treated in NICUs (Pinto et al., 2013; Nikolaou et al., 2025; Rivera-Valenzuela et al., 2026).

Compared with MRSA, transmission of methicillin-susceptible *S. aureus* in NICUs has received less attention. Previous studies indicate that *S. aureus* may be transmitted from colonized mothers, healthcare workers, other family members, contaminated surfaces, or other colonized infants, and that transmission dynamics are likely to differ between outbreaks (Harris et al., 2013; Denkel et al., 2014; Matok et al., 2021; Heigl et al., 2022; Nikolaou et al., 2025). Recent genomic surveillance studies further suggest that MSSA transmission clusters in neonatal settings may occur more frequently than previously recognized (Baker et al., 2025; Nikolaou et al., 2025; She et al., 2026). Our findings therefore add to the evidence that susceptible *S. aureus* strains can be transmitted within NICUs and may cause clinically relevant outbreaks despite the absence of antimicrobial resistance.

In a literature review, we found only a limited number of outbreaks in NICUs caused by typed MSSA. These MSSA strains belonged to sequence types ST21, ST84, and ST121 (Layer et al., 2015; Jogenfors et al., 2026; Rivera-Valenzuela et al., 2026). In the present study, the isolates forming the outbreak cluster belonged to ST15 and carried the *eta* gene. ST15 has been identified in different geographical and clinical settings and may occur as either MSSA or MRSA (Zhao et al., 2022, 2023; Bojang et al., 2024; Kawayanagi et al., 2025; Tsubaki et al., 2025). The *eta* gene has also been described in individual ST15 isolates (Mama et al., 2019; Bojang et al., 2024). Thus, ST15 represents a globally distributed lineage with variable antimicrobial resistance and virulence profiles. In the present outbreak, the combination of penicillin susceptibility, absence of *mecA* and *blaZ*, and presence of *eta* characterized the closely related isolates. Rather than representing a unique biological property of ST15 itself, these findings illustrate the considerable phenotypic and genomic diversity that may occur within this lineage.

Premature infants may be particularly susceptible to infections caused by exfoliative toxin-producing *S. aureus*. Previous data showed that antibodies against ETA were detected in 55%, 73%, and 90% of infants born at <30, 30–37, and >37 weeks of gestation, respectively (Saida et al., 2015). Consistent with this observation, gestational age in our study was significantly lower in patients with confirmed infection than in those with colonization only (median 30.6 vs. 34.8 weeks, *p* = 0.029). However, the small sample size and the presence of a term infant among the infected patients preclude conclusions regarding a causal role of gestational age. Larger studies would be required to determine whether lower gestational age independently increases the risk of symptomatic infection with ETA-producing *S. aureus*.

Recognition and reconstruction of the outbreak were complicated by several factors. Individual detections were sometimes separated by several weeks, and penicillin-susceptible *S. aureus* isolates were not routinely archived. Consequently, only four isolates from three patients during the central outbreak period were available for detailed molecular characterization. These isolates differed by only 0–7 alleles and formed a single cgMLST cluster, providing strong evidence of transmission among these cases. However, the limited availability of isolates does not allow all epidemiologically identified cases to be assigned to the ST15 outbreak clone. This is also illustrated by patient 20, whose isolate was obtained at the end of the epidemiologically defined outbreak period but belonged to a different genotype and lacked *eta*. It therefore remains possible that sporadic colonizations and infections occurred alongside transmission of the ST15 clone and that the true duration of clonal transmission was shorter than the epidemiologically defined outbreak period. These limitations highlight the value of prospective molecular surveillance. Routine storage or rapid genomic characterization of susceptible *S. aureus* isolates could facilitate earlier recognition of transmission events, particularly when individual cases are separated in time or when clinical manifestations are relatively mild. Recent studies using genomic surveillance have similarly suggested that smaller MSSA transmission clusters may otherwise remain undetected (Baker et al., 2025; Nikolaou et al., 2025; She et al., 2026).

Environmental investigations did not identify a reservoir for *S. aureus*. Because systematic identifiable staff screening was restricted by data-protection requirements, voluntary anonymous nasal screening was offered to NICU staff, together with access to mupirocin decolonization in case of a positive result. Penicillin-susceptible *S. aureus* was detected in seven staff screening samples. However, because screening was anonymous and staff isolates were not molecularly compared with patient isolates, it cannot be determined whether healthcare workers contributed to transmission. On the other hand, an outbreak of mupirocin-resistant MSSA was recently reported in a NICU (Rivera-Valenzuela et al., 2026). Nevertheless, no further outbreak-associated detections occurred after intensified infection-control measures, voluntary staff screening, and access to decolonization had been implemented. Although the observational design does not allow the effect of individual interventions to be separated, the temporal relationship and subsequent absence of further outbreak-associated cases suggest that the combined infection-control measures likely contributed to containment of the outbreak.

A further limitation is that patient-days were not available for the complete observation period because of a hospital cyberattack (Tamm, 2025). Incidence rates could therefore not be standardized to patient-days. However, the routine surveillance frequency and sampling sites remained unchanged throughout the observation period, and monthly detections of penicillin-susceptible *S. aureus* showed only weak correlations with the numbers of penicillin-resistant *S. aureus*, swabs, blood cultures, and catheter-tip cultures. These findings do not indicate a major change in sampling intensity as an explanation for the observed cluster, although residual confounding cannot be excluded.

Overall, the epidemiological data support a time-limited outbreak of penicillin-susceptible *S. aureus*, while molecular typing of the available isolates demonstrated transmission of a closely related *eta*-positive ST15 strain among the characterized isolates.

The study highlights that antimicrobial-susceptible *S. aureus* can cause clinically relevant transmission events in NICUs and that surveillance focused predominantly on antimicrobial resistance may underestimate such clusters. Molecular characterization of susceptible isolates may therefore provide important additional information when unusual increases in colonization or infection are observed.

## Data Availability

The aggregated data supporting the findings of this study are provided in the article and the Supplementary material. Individual-level clinical data are not publicly available due to patient confidentiality and data-protection requirements. Requests for access to additional anonymized data may be directed to the corresponding author, subject to applicable legal and institutional requirements. Datasets of genomic sequences are available on request and will be made available by the authors, without undue reservation.
