# Supplementary material for: Outbreak of penicillin-susceptible, eta-positive Staphylococcus aureus in a neonatal intensive care unit
Source: Front Microbiol. 2026 Oct 27;17:1946607. doi: 10.3389/fmicb.2026.1946607 (PMC13748460; doi:10.3389/fmicb.2026.1946607)
Supplement: Supplementary file 1 [file Supplementary_file_1.docx]

Supplementary Material

# Supplementary Data

## 1.1 Supplementary Figures

**Supplementary Figure 1:** Percentage of patients per month in the period 2021 – 2025 in a neonatal intensive care unit whose test material showed penicillin-sensitive *S. aureus*. The bacterial detection was differentiated according to whether there was only colonization (green bars) or infection (red bars), or whether there was an infection that could not be reliably attributed to penicillin-sensitive *S. aureus* (yellow bars). Only the initial detection was recorded for each patient. Outbreak period: Dec 2023 – May 2024. Non-outbreak period: Jan 2021 – Nov 2023 (pre-outbreak period) and Jun 2024 – Dec 2025 (Post-outbreak period)

**Supplementary Figure 2:** Gestational age in days of infants from whom penicillin-susceptible *S. aureus* was isolated between December 2023 and May 2024. Green Squares: No Infection (Colonization). Yellow Stars: Possible Infection. Red Circles: Infection.

**Supplementary Figure 3:** Number of patients per month in the period 2021 – 2025 in a neonatal intensive care unit whose test material showed penicillin-resistant but methicillin-sensitive *S. aureus*. Only the first detection per patient was recorded.

**Supplementary Figure 4:** Number of monthly swabs, blood cultures and catheter tips in the period 2021 – 2025 that were sent from a neonatal intensive care unit to the laboratory and tested there for the presence of microorganisms. The relevant data was then transferred from the laboratory software to the Hybase programme, from which the data presented was retrieved. However, the transfer of blood culture test results did not begin until October 2021.

## 1.2 Supplementary Table

**Supplementary Table 1:** Microbiological surveillance analyses of surfaces in the patient’s environment during the outbreak period.

| Environmental investigation | Number of Samples (N) | Detection of *S. aureus* |
| --- | --- | --- |
| Patient environment | 10 | No |
| Reprocessed incubators | 5 | No |
| Touch surfaces | 10 | No |
| Plaster strips | 3 | No |

## 1.3 Statistical Supplement

*Statistical Analysis of a Suspected Outbreak*

Analysis period: January 2021 – December 2025, Date of report: February 17, 2026

# 1. Background and Objective

Monthly *S. aureus* acquisition counts were recorded from January 2021 through December 2025, yielding 60 observations. Background rates were low throughout the surveillance period, with most months recording between zero and three acquisitions. A potential outbreak cluster at the end of 2023 and beginning of 2024 was identified using cumulative sum (CUSUM) analysis. The objective of this analysis is to characterise that cluster statistically, estimate the number of excess acquisitions attributable to the outbreak period, and assess the significance of the elevation in acquisition counts.

# 2. Data

The dataset comprises 60 monthly acquisition counts spanning five complete calendar years (2021–2025). Across the full period, 83 acquisitions were recorded, yielding an overall mean of 1.38 acquisitions per month. The time series is shown in Figure 1A. A sustained elevation in counts, with several months recording four to six acquisitions, is visible between December 2023 and May 2024. Outside this window, counts are generally at or below two per month, with several months recording zero acquisitions.

**Figure 1:** (A) Monthly *S. aureus* acquisition counts with the suspected outbreak window highlighted in red (December 2023 – May 2024). (B) Mean monthly acquisition counts by calendar month during the baseline period, with ±1 SEM error bars. Model A (constant mean) and Model B (1-harmonic seasonal fit) predicted curves are overlaid.

Figure 1B shows the mean count by calendar month across the baseline period (i.e., excluding the suspected outbreak). No strong systematic seasonal pattern is apparent in the raw averages, though a slight tendency toward higher counts in the first quarter of the year is visible.

# 3. Statistical Methods

## 3.1 Outbreak Window Definition

The outbreak window was defined using a cumulative sum (CUSUM) control chart applied to the monthly acquisition counts. The CUSUM statistic is defined as: Sₜ = max(0, Sₜ₋₁ + xₜ − μ₀ − k) where xₜ is the observed count in month t, μ₀ is the in-control (baseline) rate, and k is a reference value that determines the sensitivity of the chart to a shift of interest. The in-control rate μ₀ = 1.20 acquisitions per month was estimated from the pre-outbreak period (January 2021 – November 2023, n = 35 months), i.e. the data available prior to any suspected change. The reference value k was set to the optimal value for detecting a doubling of the baseline rate (μ₁ = 2μ₀ = 2.40), given by: k = (μ₁ − μ₀) / ln(μ₁ / μ₀) = 1.73. The alarm threshold was set to h = 2.3. The CUSUM first exceeded h in January 2024 (S = 3.14). The outbreak start was defined retroactively as the first month following the last reset (Sₜ = 0) before the alarm; since Sₜ = 0 in November 2023, the outbreak window begins in December 2023. The outbreak end was defined as the last month for which Sₜ ≥ h within the alarm episode; May 2024 (S = 2.41) is the last such month, with June 2024 (S = 0.48) falling below h and July 2024 resetting to zero. The CUSUM produced no false alarms during the entire baseline period (maximum S = 1.07, reached once in January 2023). The outbreak window was therefore defined as December 2023 through May 2024 (six months). All remaining months were treated as the baseline period for model estimation (n = 54 months).

## 3.2 Baseline Models

Because acquisition counts are non-negative integers and monthly incidence rates are low, Poisson regression is the natural modelling framework. The Poisson distribution assumes that the count in month t follows:

**Nₜ ~ Poisson(μₜ)**

where μₜ is the expected count. Two candidate baseline models were estimated using maximum likelihood, fitting only the 54 baseline months:

Model A — Intercept only (no seasonal adjustment):

**log(μₜ) = β₀**

This model estimates a single constant expected rate λ = e^β₀ for all months. It assumes that any month-to-month variation in the baseline is purely random (Poisson noise).

Model B — One-harmonic seasonal adjustment:

**log(μₜ) = β₀ + β₁ · sin(2πm/12) + β₂ · cos(2πm/12)**

where m denotes the calendar month (0 = January, …, 11 = December). The sine and cosine terms together form a single annual harmonic, allowing the expected rate to vary smoothly across the calendar year. This is the most parsimonious parametric form of seasonality (two extra parameters). A likelihood ratio test was used to assess whether the seasonal terms provided a statistically significant improvement over Model A.

## 3.3 Goodness of Fit

Model fit was assessed using the Akaike Information Criterion (AIC) and Bayesian Information Criterion (BIC), both of which penalise model complexity. The Pearson dispersion statistic — the sum of squared Pearson residuals divided by the residual degrees of freedom — was computed to assess whether the data satisfied the Poisson assumption of equal mean and variance. A value close to 1 indicates adequate Poisson fit; values substantially above 1 indicate overdispersion, which would require a negative-binomial or quasi-Poisson correction.

## 3.4 Excess Case Estimation and Significance Testing

For each month within the outbreak window, the expected count μₜ was obtained by projecting the fitted baseline model forward (i.e., applying the estimated parameters to the calendar month of interest). The excess count was defined as the difference between the observed count and the model-projected expected count:

**Excessₜ = Nₜ − μₜ**

A one-sided exact Poisson test was applied to each outbreak month individually: the p-value is the probability of observing a count at least as large as Nₜ under the null hypothesis that the true rate equals μₜ, i.e. P(X ≥ Nₜ | X ~ Poisson(μₜ)). For the overall outbreak period, the same test was applied to the sum of observed counts against the sum of expected counts across all six months. A 95% confidence interval for the total excess was derived using a Wald approximation: excess ± 1.96 · √(total expected).

All analyses were performed in Python 3 using NumPy and SciPy. Statistical significance was assessed at α = 0.05.

# 4. Results

## 4.1 Model Fit

Table 1 summarises the estimated parameters and goodness-of-fit statistics for both models, based on the 54 baseline months.

Both models estimated a baseline rate of approximately 1.17 cases per month. The Pearson dispersion statistics (0.865 for Model A and 0.845 for Model B) are both close to and below 1, confirming that there is no overdispersion in the baseline data and that standard Poisson inference is appropriate. The likelihood ratio test comparing Model B to Model A yielded χ²(2) = 4.24, p = 0.120, indicating that the seasonal harmonic terms do not provide a statistically significant improvement. The AIC is virtually identical for both models (108.58 vs. 108.33), while the BIC strongly favours the simpler Model A (110.57 vs. 114.30). On the basis of these criteria, Model A is the preferred parsimonious model.

This statistical preference for Model A is further supported by clinical and epidemiological reasoning. Hospital-acquired colonization of *S. aureus* is not transmitted via a seasonal environmental or vector-dependent route. The bacterial colonisation of newborns occurs essentially without a predictable annual periodicity via bacterial transfer from mother to child. There is therefore no medical basis to expect a systematic seasonal pattern in these colonisation counts. The absence of statistically significant seasonality in the data is consistent with this expectation. From this point forward, all analyses are based exclusively on Model A.

**Table 1:** Comparison of baseline Poisson models fitted to the 54 non-outbreak months.

| **Parameter** | **Model A** |  | **Model B** |  |
| --- | --- | --- | --- | --- |
|  | **Estimate** |  | **Estimate** |  |
| Intercept (β₀) | log λ = 0.154 | λ = 1.167 | log λ = 0.147 | λ = 1.158 |
| Sine coefficient (β₁) | — | — | 0.228 |  |
| Cosine coefficient (β₂) | — | — | 0.289 |  |
| Fitted mean (baseline) | 1.17 cases/mo |  | 1.17 cases/mo | (at peak season) |
| Pearson dispersion | 0.865 | (< 1) | 0.845 | (< 1) |
| Log-likelihood | −53.29 |  | −51.17 |  |
| AIC | 108.58 |  | 108.33 |  |
| BIC | 110.57 |  | 114.30 |  |

## 4.2 Excess Cases During the Outbreak Window

Figure 2A shows the observed monthly counts together with the expected rate from Model A across the full five-year period. The sustained elevation within the outbreak window is visually striking relative to both the surrounding baseline and the model-projected rate.

Figure 2B shows the excess acquisitions per month within the outbreak window, and Table 2 provides the corresponding one-sided Poisson p-values.

**Figure 2:** (A) Observed acquisition counts (bars) vs. Model A projected expected rate (line) for the full surveillance period. The outbreak window (December 2023 – May 2024) is shaded. (B) Excess acquisitions per month during the outbreak window under Model A. Months with statistically significant excess are annotated with their one-sided p-value.

**Table 2:** Month-by-month excess acquisitions and one-sided Poisson p-values — Model A (intercept only, expected = 1.17/month).

| **Month** | **Observed** | **Expected** | **Excess** | **p-value** | **Sig.** |
| --- | --- | --- | --- | --- | --- |
| Dec 2023 | 4 | 1.17 | +2.83 | 0.031 | * |
| Jan 2024 | 5 | 1.17 | +3.83 | 0.007 | ** |
| Feb 2024 | 1 | 1.17 | −0.17 | 0.690 |  |
| Mar 2024 | 2 | 1.17 | +0.83 | 0.326 |  |
| Apr 2024 | 5 | 1.17 | +3.83 | 0.007 | ** |
| May 2024 | 3 | 1.17 | +1.83 | 0.114 |  |
| **TOTAL** | **20** | **7.00** | **+13.00** | **< 0.001** | ******* |

Three individual months show statistically significant excess: December 2023 (p = 0.031), January 2024 (p = 0.007), and April 2024 (p = 0.007). February, March, and May 2024 did not show individually significant excess, suggesting that the outbreak signal was concentrated in three distinct months rather than uniformly elevated throughout the window.

The total observed count during the six-month window was 20 acquisitions against a Model A projection of 7.0, yielding a highly significant overall excess:

• Model A: excess = +13.0 acquisitions (95% CI: 7.8 – 18.2), p < 0.001

Model A thus attributes approximately 13 excess acquisitions to the outbreak period (95% CI: 7.8 – 18.2), representing an approximately 3-fold increase over the expected baseline rate.

## 4.3 Infection Status of Detections During the Outbreak Window

Each *S. aureus* detection was classified as a confirmed infection (“Yes”), an uncertain case (“Maybe”), or a colonisation without infection evidence (“No”). Across the 54 baseline months, 16 confirmed infections and 5 uncertain cases were recorded among 63 total detections, yielding a baseline infection rate of 0.30 confirmed infections per month (0.39/month including uncertain cases). During the six-month outbreak window, 10 confirmed infections and 2 uncertain cases occurred among 20 total detections.

Applying the same Poisson framework as in Section 4.2, the absolute number of confirmed infections during the outbreak window was highly significantly elevated: 10 observed versus 1.78 expected (p < 0.001), corresponding to a 5.6-fold increase and an excess of +8.2 infections (95% CI: 5.6 – 10.8). The result is virtually identical when uncertain cases are included: 12 observed versus 2.33 expected (p < 0.001), a 5.1-fold increase, excess +9.7 (95% CI: 6.7 – 12.7). The outbreak was therefore not merely an increase in colonisation: the absolute number of infections rose dramatically and significantly regardless of how the ambiguous cases are classified.

A complementary question is whether infections also made up a larger proportion of detections during the outbreak, that is, whether the composition of cases shifted toward true infections, rather than simply reflecting a proportional rise in all detections. The confirmed infection proportion increased from 25.4% at baseline to 50.0% during the outbreak window (Figure 3). A one-sided Fisher’s exact test yields an odds ratio of 2.94, with p = 0.039 for confirmed infections alone and p = 0.032 when uncertain cases are included. This result indicates a significant shift in the composition of detections toward true infections. Taken together, the two analyses consistently indicate that the outbreak was driven by a genuine and substantial increase in infections, not by colonisation alone.

**Figure 3:** Stacked bar chart showing the proportion of *S. aureus* detections classified as confirmed infections (Yes), uncertain cases (Maybe), and colonisation without infection (No), for the baseline period and the outbreak window (December 2023 – May 2024). The confirmed infection proportion increased from 25.4% to 50.0%. Fisher’s exact test: OR = 2.94, p = 0.032 (including uncertain cases), p = 0.039 (confirmed infections only).

# 5. Conclusions

The statistical analysis supports the clinical hypothesis that an outbreak occurred centred on the period December 2023 to May 2024. The key findings are:

1. The baseline *S. aureus* acquisition rate was stable at approximately 1.2 acquisitions per month throughout 2021–2023 and again from July 2024 onwards, with no evidence of a significant seasonal trend.

2. A Poisson regression model (Model A, constant mean) fitted to the 54 non-outbreak months provides an expected baseline rate against which the outbreak period can be compared. The Pearson dispersion statistic confirms that standard Poisson assumptions are met (no overdispersion). Seasonality was not statistically significant, and is not expected for neonatal *S. aureus* acquisitions; Model A is therefore the sole model used for inference.

3. Within the outbreak window, the overall count of 20 acquisitions was significantly higher than expected under Model A (p < 0.001). Three individual months — December 2023, January 2024, and April 2024 — showed individually significant elevations.

4. Approximately 13 excess acquisitions can be attributed to the outbreak period (95% CI: 7.8 – 18.2), representing an approximately 3-fold increase over the expected baseline rate.

5. The outbreak was not merely an increase in colonisation. The absolute number of confirmed infections rose by a factor of 5.6 (10 observed vs. 1.78 expected, p < 0.001). There is also evidence of a compositional shift: the proportion of detections classified as infections increased from 25.4% at baseline to 50.0% during the outbreak (Fisher’s exact test OR = 2.94, p = 0.032 including uncertain cases, p = 0.039 for confirmed infections only).

# Appendix: Statistical Notes

## Poisson Distribution

The Poisson distribution is a discrete probability distribution appropriate for modelling counts of rare, independent events occurring at a constant rate over a fixed observation period. Its probability mass function is:

**P(X = k) = e^(−μ) · μᵏ / k!**

where μ is both the mean and the variance of the distribution. The equal-mean-variance property makes the Pearson dispersion statistic a natural goodness-of-fit diagnostic: systematic overdispersion (dispersion >> 1) would indicate that a negative-binomial model is preferable.

## One-Sided Exact Poisson Test

For a given month with observed count N and model-expected count μ, the one-sided p-value for an excess is computed as the tail probability of the Poisson distribution:

**p = P(X ≥ N | X ~ Poisson(μ)) = 1 − P(X ≤ N−1 | X ~ Poisson(μ))**

This is an exact test (not an approximation), which is important given the small counts involved. The same logic is applied to the summed counts over the entire outbreak window.

## Note on Significance Levels

Because six months were tested individually, a Bonferroni-corrected threshold of α = 0.05/6 ≈ 0.008 could be applied for the per-month tests. Under this stricter threshold, January 2024 (p = 0.007) and April 2024 (p = 0.007) both fall just below the corrected threshold of 0.008, and well below the uncorrected threshold of 0.05. December 2023 (p = 0.031) is significant at the uncorrected level only. The overall test across the full window is not subject to this concern, as it is a single pre-specified hypothesis. The outbreak cluster as a whole is unambiguously significant regardless of the multiple-comparison adjustment used.
